# Poly[[(μ-3-amino­pyrazine-2-carboxyl­ato-κ^3^ *N* ^1^,*O*:*O*′)diaqua­(μ-oxalato-κ^4^ *O* ^1^,*O* ^2^:*O* ^1′^,*O* ^2′^)lanthanum(III)] monohydrate]

**DOI:** 10.1107/S1600536811034404

**Published:** 2011-08-27

**Authors:** Shan Gao, Seik Weng Ng

**Affiliations:** a Key Laboratory of Functional Inorganic Material Chemistry, Ministry of Education, Heilongjiang University, Harbin 150080, People’s Republic of China; b Department of Chemistry, University of Malaya, 50603 Kuala Lumpur, Malaysia; c Chemistry Department, Faculty of Science, King Abdulaziz University, PO Box 80203 Jeddah, Saudi Arabia

## Abstract

The water-coordinated La^III^ atom in the title compound, {[La(C_5_H_4_N_3_O_2_)(C_2_O_4_)(H_2_O)_2_]·H_2_O}_*n*_, is *N*,*O*-chelated by a 3-amino­pyrazine-2-carboxyl­ate ion; this ion links adjacent metal atoms to form a chain parallel to [010]. The oxalate ion serves as a bis-bidentate chelate that links adjacent metal atoms to form a chain parallel to [001]. The two bridging ions give rise to a layer motif parallel to (100) in which the La^III^ atom exists in a distorted tricapped trigonal prismatic geometry. Extensive hydrogen bonding between the constituents stabilizes the structure.

## Related literature

For a related structure, see: Leciejewicz *et al.* (2004 ▶). For pyrazine­carb­oxy­lic acid decomposition with subsequent oxalate formation, which has been documented in other lanthanum systems, see: Li *et al.* (2006 ▶).
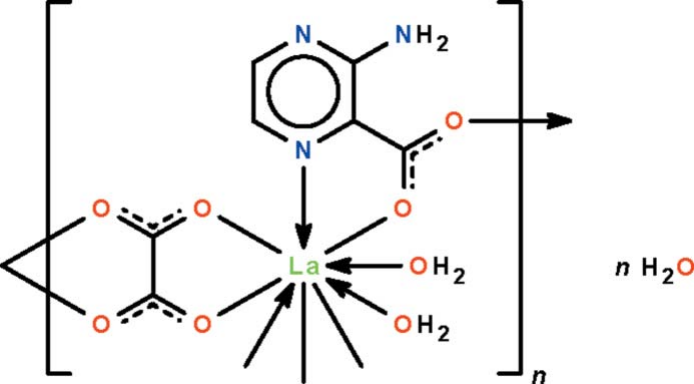


## Experimental

### 

#### Crystal data

[La(C_5_H_4_N_3_O_2_)(C_2_O_4_)(H_2_O)_2_]·H_2_O
*M* *_r_* = 419.09
Monoclinic, 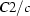

*a* = 18.2193 (5) Å
*b* = 10.5507 (3) Å
*c* = 13.1307 (5) Å
β = 105.292 (1)°
*V* = 2434.70 (13) Å^3^
*Z* = 8
Mo *K*α radiation
μ = 3.56 mm^−1^
*T* = 293 K
0.14 × 0.12 × 0.08 mm

#### Data collection

Rigaku RAXIS-RAPID IP diffractometer
Absorption correction: multi-scan (*ABSCOR*; Higashi, 1995 ▶) *T*_min_ = 0.636, *T*_max_ = 0.764
11571 measured reflections
2780 independent reflections
2408 reflections with *I* > 2σ(*I*)
*R*_int_ = 0.038

#### Refinement

*R*[*F* ^2^ > 2σ(*F* ^2^)] = 0.025
*wR*(*F* ^2^) = 0.064
*S* = 1.03
2780 reflections
213 parameters
11 restraints
H atoms treated by a mixture of independent and constrained refinement
Δρ_max_ = 1.19 e Å^−3^
Δρ_min_ = −0.90 e Å^−3^

### 

Data collection: *RAPID-AUTO* (Rigaku, 1998 ▶); cell refinement: *RAPID-AUTO*; data reduction: *CrystalClear* (Rigaku/MSC, 2002 ▶); program(s) used to solve structure: *SHELXS97* (Sheldrick, 2008 ▶); program(s) used to refine structure: *SHELXL97* (Sheldrick, 2008 ▶); molecular graphics: *X-SEED* (Barbour, 2001 ▶); software used to prepare material for publication: *publCIF* (Westrip, 2010 ▶).

## Supplementary Material

Crystal structure: contains datablock(s) global, I. DOI: 10.1107/S1600536811034404/qk2018sup1.cif

Structure factors: contains datablock(s) I. DOI: 10.1107/S1600536811034404/qk2018Isup2.hkl

Additional supplementary materials:  crystallographic information; 3D view; checkCIF report

## Figures and Tables

**Table 1:** Hydrogen-bond geometry (Å, °)

*D*—H⋯*A*	*D*—H	H⋯*A*	*D*⋯*A*	*D*—H⋯*A*
O1*W*—H11⋯O6^i^	0.84 (1)	1.89 (1)	2.720 (3)	169 (3)
O1*W*—H12⋯N2^ii^	0.84 (1)	2.00 (1)	2.842 (3)	175 (3)
O2*W*—H21⋯O5^iii^	0.84 (1)	1.95 (1)	2.787 (3)	175 (4)
O2*W*—H22⋯O3*W*	0.84 (1)	2.16 (2)	2.908 (4)	148 (4)
O3*W*—H31⋯O2*W*^iv^	0.84 (1)	2.19 (1)	3.017 (4)	165 (4)
O3*W*—H32⋯N3^iii^	0.84 (1)	2.33 (2)	3.152 (5)	165 (4)
N3—H1⋯O2	0.88 (1)	2.06 (3)	2.711 (3)	130 (3)
N3—H2⋯O3^v^	0.88 (1)	2.10 (1)	2.967 (3)	167 (3)

Symmetry codes: (i) 
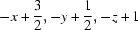
; (ii) 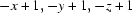; (iii) 
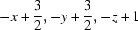
; (iv) 
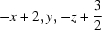
; (v) 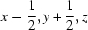.

**Fig. 1.**
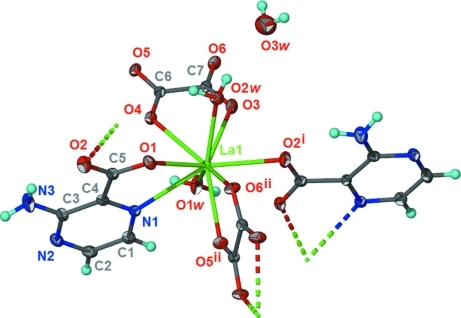
Thermal ellipsoid plot (Barbour, 2001) of a portion of the layer structure of La(H2O)2(C2O4)(C5H4N3O2).H2O at the 50% probability level; H atoms are drawn as spheres of arbitrary radius.

## supplementary crystallographic information

### Comment

The chelating ability of the 3-aminopyrazine-2-carboxylate anion is probably similar to that of the pyrazine-2-carboxylate anion, and the crystal structures of a number of lanthanum carboxylates have been reported. Hydrated lanthanum tris(pyrazine-2-carboxylate) adopts a chain motif (Leciejewicz *et al.*, 2004). The additional amino substitution in the 3-aminopyrazine-2-carboxylate should be expected to consolidate the crystal structure of the title lanthanum derivative through extensive hydrogen bonding. The water-coordinated La^III^ atom in La(H_2_O)_2_(C_2_O_4_)(C_5_H_4_N_3_O_2_).H_2_O (Scheme I, Fig. 1) is *N*,*O*-chelated by an 3-aminopyrazine-2-carboxylate ion; this ion links adjacent metal atoms to form a chain parallel to [010]. The presence of an oxalate ion is explained by the decomposition of 3-aminopyrazine-2-carboxylic acid; the oxalate ion serves as a bis-bidentate chelate that links adjacent metal atoms. The two bridging ions give rise to a layer motif parallel to [100] in which the La^III^ atom exists in a nine-coordinate environment. The geometry is best described as a distorted tricapped trigonal prism. The upper prism triangle is made up of the atoms O1, O4 and O2*w*, and the lower prism triangle by the atoms O2, O5 and O1*w*.

The layers interact with the lattice water molecules to generate a three-dimensional hydrogen-bonded network (Table 1).

### Experimental

Lanthanum nitrate hexahydrate (0.5 mmol) and 3-aminopyrazine-2-carboxylic acid (2 mmol) were dissolved in water (15 ml). The solution was sealed in a 25 ml Teflon-lined stainless steel bomb and held at 443 K for 3 d. The bomb was gradually cooled to room temperature, and colorless prismatic crystals were obtained.

### Refinement

Carbon- and nitrogen-bound H atoms were placed in calculated positions (C—H 0.93 Å, N—H 0.88 Å) and were included in the refinement in the riding model approximation, with *U*_iso_(H) set to 1.2*U*_eq_(C,N). The water H atoms were located in a difference Fourier map, and were refined with distance restraints of O—H 0.84 (1) Å and H···H 1.37 (1) Å; their temperature factors were tied by a factor of 1.5 times.

The final difference Fourier map had the largest peaks and holes in the vicinity of La1.

### Crystal data

**Table:**

[La(C_5_H_4_N_3_O_2_)(C_2_O_4_)(H_2_O)_2_]·H_2_O	*F*(000) = 1616
*M**_r_* = 419.09	*D*_x_ = 2.287 Mg m^−^^3^
Monoclinic, *C*2/*c*	Mo *K*α radiation, λ = 0.71073 Å
Hall symbol: -C 2yc	Cell parameters from 8439 reflections
*a* = 18.2193 (5) Å	θ = 3.2–27.4°
*b* = 10.5507 (3) Å	µ = 3.56 mm^−^^1^
*c* = 13.1307 (5) Å	*T* = 293 K
β = 105.292 (1)°	Prism, colourless
*V* = 2434.70 (13) Å^3^	0.14 × 0.12 × 0.08 mm
*Z* = 8	

### Data collection

**Table:**

Rigaku RAXIS-RAPID IP diffractometer	2780 independent reflections
Radiation source: fine-focus sealed tube	2408 reflections with *I* > 2σ(*I*)
graphite	*R*_int_ = 0.038
ω scans	θ_max_ = 27.4°, θ_min_ = 3.2°
Absorption correction: multi-scan (*ABSCOR*; Higashi, 1995)	*h* = −22→23
*T*_min_ = 0.636, *T*_max_ = 0.764	*k* = −13→11
11571 measured reflections	*l* = −17→17

### Refinement

**Table:**

Refinement on *F*^2^	Primary atom site location: structure-invariant direct methods
Least-squares matrix: full	Secondary atom site location: difference Fourier map
*R*[*F*^2^ > 2σ(*F*^2^)] = 0.025	Hydrogen site location: inferred from neighbouring sites
*wR*(*F*^2^) = 0.064	H atoms treated by a mixture of independent and constrained refinement
*S* = 1.03	*w* = 1/[σ^2^(*F*_o_^2^) + (0.0366*P*)^2^ + 0.9654*P*] where *P* = (*F*_o_^2^ + 2*F*_c_^2^)/3
2780 reflections	(Δ/σ)_max_ = 0.001
213 parameters	Δρ_max_ = 1.19 e Å^−^^3^
11 restraints	Δρ_min_ = −0.90 e Å^−^^3^

### Fractional atomic coordinates and isotropic or equivalent isotropic displacement parameters (Å^2^)

**Table:**

	*x*	*y*	*z*	*U*_iso_*/*U*_eq_	
La1	0.753537 (8)	0.505715 (13)	0.708865 (11)	0.01578 (8)	
O1	0.70771 (10)	0.72700 (18)	0.73633 (16)	0.0229 (4)	
O3	0.82152 (11)	0.4381 (2)	0.56948 (15)	0.0265 (5)	
O4	0.70436 (11)	0.60558 (18)	0.52529 (15)	0.0232 (4)	
O5	0.70189 (11)	0.59526 (17)	0.35400 (15)	0.0215 (4)	
O6	0.81026 (11)	0.41769 (18)	0.39750 (15)	0.0237 (4)	
O1W	0.67446 (12)	0.33869 (19)	0.59002 (18)	0.0301 (5)	
H11	0.6840 (18)	0.2609 (12)	0.590 (3)	0.046 (11)*	
H12	0.6393 (18)	0.356 (3)	0.536 (2)	0.064 (13)*	
O2W	0.86289 (12)	0.66714 (19)	0.70250 (17)	0.0273 (5)	
H21	0.8424 (19)	0.7373 (19)	0.682 (3)	0.053 (12)*	
H22	0.8870 (19)	0.643 (3)	0.660 (2)	0.052 (12)*	
O2	0.62477 (11)	0.88451 (18)	0.70103 (17)	0.0283 (5)	
O3W	0.99478 (18)	0.6325 (3)	0.6200 (3)	0.0556 (7)	
H31	1.0385 (11)	0.644 (4)	0.661 (2)	0.075 (17)*	
H32	0.999 (2)	0.628 (4)	0.5577 (12)	0.073 (16)*	
N1	0.59859 (12)	0.5541 (2)	0.67230 (18)	0.0188 (5)	
N2	0.44670 (13)	0.6197 (2)	0.5937 (2)	0.0241 (5)	
N3	0.47703 (15)	0.8320 (3)	0.6050 (3)	0.0338 (7)	
H1	0.5096 (15)	0.893 (2)	0.630 (3)	0.033 (10)*	
H2	0.4284 (8)	0.851 (3)	0.593 (3)	0.043 (10)*	
C1	0.54410 (17)	0.4653 (3)	0.6480 (2)	0.0233 (6)	
H1A	0.5572	0.3801	0.6577	0.028*	
C2	0.4688 (2)	0.4990 (2)	0.6088 (3)	0.0256 (7)	
H2A	0.4321	0.4355	0.5924	0.031*	
C3	0.50011 (15)	0.7099 (3)	0.6213 (2)	0.0204 (6)	
C4	0.57805 (15)	0.6755 (2)	0.6627 (2)	0.0176 (5)	
C5	0.64079 (15)	0.7689 (3)	0.7022 (2)	0.0196 (6)	
C6	0.72721 (15)	0.5654 (2)	0.4499 (2)	0.0177 (5)	
C7	0.79244 (16)	0.4649 (3)	0.4748 (2)	0.0180 (5)	

### Atomic displacement parameters (Å^2^)

**Table:**

	*U*^11^	*U*^22^	*U*^33^	*U*^12^	*U*^13^	*U*^23^
La1	0.01427 (11)	0.02279 (11)	0.00982 (11)	0.00134 (6)	0.00236 (7)	0.00027 (5)
O1	0.0147 (10)	0.0293 (10)	0.0226 (11)	−0.0015 (9)	0.0012 (8)	−0.0039 (8)
O3	0.0269 (11)	0.0386 (12)	0.0137 (10)	0.0120 (10)	0.0048 (8)	0.0028 (9)
O4	0.0259 (10)	0.0301 (10)	0.0150 (10)	0.0091 (9)	0.0081 (8)	0.0023 (8)
O5	0.0249 (10)	0.0275 (10)	0.0117 (10)	0.0057 (8)	0.0038 (8)	0.0036 (8)
O6	0.0312 (11)	0.0256 (10)	0.0149 (10)	0.0068 (9)	0.0072 (8)	0.0002 (8)
O1W	0.0312 (12)	0.0211 (10)	0.0297 (13)	0.0042 (10)	−0.0066 (10)	−0.0027 (9)
O2W	0.0276 (11)	0.0256 (11)	0.0309 (13)	0.0028 (10)	0.0117 (10)	0.0026 (9)
O2	0.0225 (10)	0.0244 (10)	0.0337 (13)	−0.0033 (9)	−0.0001 (9)	−0.0059 (9)
O3W	0.0518 (18)	0.071 (2)	0.0455 (19)	0.0010 (17)	0.0152 (15)	−0.0004 (16)
N1	0.0172 (11)	0.0232 (12)	0.0160 (12)	0.0003 (10)	0.0045 (9)	0.0008 (9)
N2	0.0165 (11)	0.0327 (13)	0.0223 (13)	−0.0017 (11)	0.0038 (10)	−0.0022 (10)
N3	0.0189 (14)	0.0286 (14)	0.0486 (19)	0.0028 (12)	−0.0005 (13)	−0.0035 (13)
C1	0.0224 (15)	0.0234 (13)	0.0242 (16)	−0.0008 (13)	0.0064 (12)	0.0004 (12)
C2	0.0213 (16)	0.0301 (17)	0.0256 (18)	−0.0086 (12)	0.0067 (13)	−0.0006 (11)
C3	0.0184 (13)	0.0275 (14)	0.0150 (14)	0.0026 (12)	0.0038 (11)	−0.0032 (11)
C4	0.0163 (13)	0.0233 (13)	0.0129 (13)	−0.0031 (11)	0.0032 (10)	−0.0025 (10)
C5	0.0195 (14)	0.0264 (14)	0.0133 (14)	0.0007 (12)	0.0049 (11)	−0.0008 (11)
C6	0.0180 (13)	0.0189 (13)	0.0163 (14)	−0.0004 (12)	0.0044 (10)	0.0012 (10)
C7	0.0163 (14)	0.0225 (12)	0.0144 (14)	0.0003 (12)	0.0029 (11)	0.0002 (11)

### Geometric parameters (Å, °)

**Table:**

La1—O1W	2.536 (2)	O2W—H22	0.838 (10)
La1—O1	2.5371 (19)	O2—C5	1.254 (3)
La1—O6^i^	2.5507 (19)	O2—La1^iv^	2.5627 (19)
La1—O2^ii^	2.5627 (19)	O3W—H31	0.844 (10)
La1—O4	2.5659 (19)	O3W—H32	0.843 (10)
La1—O3	2.5668 (19)	N1—C4	1.331 (3)
La1—O5^i^	2.5687 (18)	N1—C1	1.341 (4)
La1—O2W	2.639 (2)	N2—C2	1.334 (3)
La1—N1	2.783 (2)	N2—C3	1.341 (4)
O1—C5	1.263 (3)	N3—C3	1.355 (4)
O3—C7	1.248 (3)	N3—H1	0.880 (10)
O4—C6	1.246 (3)	N3—H2	0.880 (10)
O5—C6	1.261 (3)	C1—C2	1.379 (5)
O5—La1^iii^	2.5687 (18)	C1—H1A	0.9300
O6—C7	1.248 (3)	C2—H2A	0.9300
O6—La1^iii^	2.5507 (19)	C3—C4	1.427 (4)
O1W—H11	0.838 (10)	C4—C5	1.495 (4)
O1W—H12	0.841 (10)	C6—C7	1.561 (4)
O2W—H21	0.842 (10)		
			
O1W—La1—O1	124.90 (6)	C6—O5—La1^iii^	121.85 (17)
O1W—La1—O6^i^	146.79 (7)	C7—O6—La1^iii^	122.53 (17)
O1—La1—O6^i^	68.57 (6)	La1—O1W—H11	126 (2)
O1W—La1—O2^ii^	102.67 (6)	La1—O1W—H12	123 (2)
O1—La1—O2^ii^	132.12 (6)	H11—O1W—H12	109.1 (17)
O6^i^—La1—O2^ii^	68.29 (7)	La1—O2W—H21	108 (2)
O1W—La1—O4	72.92 (7)	La1—O2W—H22	111 (3)
O1—La1—O4	73.09 (6)	H21—O2W—H22	108.7 (17)
O6^i^—La1—O4	137.21 (6)	C5—O2—La1^iv^	108.04 (16)
O2^ii^—La1—O4	133.14 (6)	H31—O3W—H32	108.1 (17)
O1W—La1—O3	70.77 (7)	C4—N1—C1	118.5 (2)
O1—La1—O3	126.58 (7)	C4—N1—La1	116.11 (17)
O6^i^—La1—O3	129.22 (6)	C1—N1—La1	124.51 (19)
O2^ii^—La1—O3	70.67 (7)	C2—N2—C3	117.9 (2)
O4—La1—O3	63.83 (6)	C3—N3—H1	119 (2)
O1W—La1—O5^i^	84.75 (7)	C3—N3—H2	120 (2)
O1—La1—O5^i^	94.45 (6)	H1—N3—H2	117 (3)
O6^i^—La1—O5^i^	62.92 (6)	N1—C1—C2	120.7 (3)
O2^ii^—La1—O5^i^	83.81 (6)	N1—C1—H1A	119.7
O4—La1—O5^i^	139.62 (6)	C2—C1—H1A	119.7
O3—La1—O5^i^	138.92 (6)	N2—C2—C1	122.2 (3)
O1W—La1—O2W	137.74 (7)	N2—C2—H2A	118.9
O1—La1—O2W	71.95 (6)	C1—C2—H2A	118.9
O6^i^—La1—O2W	73.16 (6)	N2—C3—N3	117.4 (2)
O2^ii^—La1—O2W	76.60 (6)	N2—C3—C4	120.1 (2)
O4—La1—O2W	77.57 (7)	N3—C3—C4	122.6 (3)
O3—La1—O2W	69.30 (7)	N1—C4—C3	120.5 (2)
O5^i^—La1—O2W	135.93 (6)	N1—C4—C5	115.5 (2)
O1W—La1—N1	68.62 (7)	C3—C4—C5	123.9 (2)
O1—La1—N1	59.92 (6)	O2—C5—O1	123.0 (2)
O6^i^—La1—N1	104.01 (6)	O2—C5—C4	118.9 (2)
O2^ii^—La1—N1	152.36 (7)	O1—C5—C4	118.1 (2)
O4—La1—N1	71.07 (6)	O4—C6—O5	126.7 (3)
O3—La1—N1	125.89 (6)	O4—C6—C7	117.4 (2)
O5^i^—La1—N1	69.55 (6)	O5—C6—C7	115.9 (2)
O2W—La1—N1	128.03 (7)	O3—C7—O6	125.9 (3)
C5—O1—La1	126.49 (17)	O3—C7—C6	117.6 (2)
C7—O3—La1	119.28 (17)	O6—C7—C6	116.5 (2)
C6—O4—La1	120.00 (17)		
			
O1W—La1—O1—C5	7.2 (2)	O3—La1—N1—C1	69.9 (2)
O6^i^—La1—O1—C5	−138.1 (2)	O5^i^—La1—N1—C1	−66.4 (2)
O2^ii^—La1—O1—C5	−165.2 (2)	O2W—La1—N1—C1	160.6 (2)
O4—La1—O1—C5	61.3 (2)	C4—N1—C1—C2	3.3 (4)
O3—La1—O1—C5	98.2 (2)	La1—N1—C1—C2	−165.7 (2)
O5^i^—La1—O1—C5	−79.5 (2)	C3—N2—C2—C1	−2.2 (5)
O2W—La1—O1—C5	143.4 (2)	N1—C1—C2—N2	−0.3 (5)
N1—La1—O1—C5	−16.2 (2)	C2—N2—C3—N3	179.7 (3)
O1W—La1—O3—C7	67.4 (2)	C2—N2—C3—C4	1.6 (4)
O1—La1—O3—C7	−52.4 (2)	C1—N1—C4—C3	−3.8 (4)
O6^i^—La1—O3—C7	−143.4 (2)	La1—N1—C4—C3	166.12 (19)
O2^ii^—La1—O3—C7	179.0 (2)	C1—N1—C4—C5	173.5 (2)
O4—La1—O3—C7	−12.6 (2)	La1—N1—C4—C5	−16.5 (3)
O5^i^—La1—O3—C7	124.2 (2)	N2—C3—C4—N1	1.4 (4)
O2W—La1—O3—C7	−98.5 (2)	N3—C3—C4—N1	−176.6 (3)
N1—La1—O3—C7	24.1 (2)	N2—C3—C4—C5	−175.7 (3)
O1W—La1—O4—C6	−66.9 (2)	N3—C3—C4—C5	6.3 (4)
O1—La1—O4—C6	157.1 (2)	La1^iv^—O2—C5—O1	−4.6 (3)
O6^i^—La1—O4—C6	130.02 (19)	La1^iv^—O2—C5—C4	174.72 (19)
O2^ii^—La1—O4—C6	24.6 (2)	La1—O1—C5—O2	−166.0 (2)
O3—La1—O4—C6	9.60 (19)	La1—O1—C5—C4	14.7 (3)
O5^i^—La1—O4—C6	−126.34 (19)	N1—C4—C5—O2	−176.0 (2)
O2W—La1—O4—C6	82.4 (2)	C3—C4—C5—O2	1.2 (4)
N1—La1—O4—C6	−139.6 (2)	N1—C4—C5—O1	3.3 (4)
O1W—La1—N1—C4	−143.34 (19)	C3—C4—C5—O1	−179.4 (3)
O1—La1—N1—C4	16.16 (17)	La1—O4—C6—O5	171.5 (2)
O6^i^—La1—N1—C4	70.73 (18)	La1—O4—C6—C7	−6.8 (3)
O2^ii^—La1—N1—C4	140.54 (18)	La1^iii^—O5—C6—O4	−177.5 (2)
O4—La1—N1—C4	−64.83 (18)	La1^iii^—O5—C6—C7	0.8 (3)
O3—La1—N1—C4	−99.37 (19)	La1—O3—C7—O6	−164.8 (2)
O5^i^—La1—N1—C4	124.31 (19)	La1—O3—C7—C6	14.4 (3)
O2W—La1—N1—C4	−8.7 (2)	La1^iii^—O6—C7—O3	−174.9 (2)
O1W—La1—N1—C1	25.9 (2)	La1^iii^—O6—C7—C6	5.8 (3)
O1—La1—N1—C1	−174.6 (2)	O4—C6—C7—O3	−5.2 (4)
O6^i^—La1—N1—C1	−120.0 (2)	O5—C6—C7—O3	176.4 (2)
O2^ii^—La1—N1—C1	−50.2 (3)	O4—C6—C7—O6	174.1 (2)
O4—La1—N1—C1	104.4 (2)	O5—C6—C7—O6	−4.3 (4)

Symmetry codes: (i) *x*, −*y*+1, *z*+1/2; (ii) −*x*+3/2, *y*−1/2, −*z*+3/2; (iii) *x*, −*y*+1, *z*−1/2; (iv) −*x*+3/2, *y*+1/2, −*z*+3/2.

### Hydrogen-bond geometry (Å, °)

**Table:**

*D*—H···*A*	*D*—H	H···*A*	*D*···*A*	*D*—H···*A*
O1W—H11···O6^v^	0.84 (1)	1.89 (1)	2.720 (3)	169 (3)
O1W—H12···N2^vi^	0.84 (1)	2.00 (1)	2.842 (3)	175 (3)
O2W—H21···O5^vii^	0.84 (1)	1.95 (1)	2.787 (3)	175 (4)
O2W—H22···O3W	0.84 (1)	2.16 (2)	2.908 (4)	148 (4)
O3W—H31···O2W^viii^	0.84 (1)	2.19 (1)	3.017 (4)	165 (4)
O3W—H32···N3^vii^	0.84 (1)	2.33 (2)	3.152 (5)	165 (4)
N3—H1···O2	0.88 (1)	2.06 (3)	2.711 (3)	130 (3)
N3—H2···O3^ix^	0.88 (1)	2.10 (1)	2.967 (3)	167 (3)

Symmetry codes: (v) −*x*+3/2, −*y*+1/2, −*z*+1; (vi) −*x*+1, −*y*+1, −*z*+1; (vii) −*x*+3/2, −*y*+3/2, −*z*+1; (viii) −*x*+2, *y*, −*z*+3/2; (ix) *x*−1/2, *y*+1/2, *z*.
